# Intervention, individual, and contextual determinants to high adherence to structured family-centered rounds: a national multi-site mixed methods study

**DOI:** 10.1186/s43058-022-00322-1

**Published:** 2022-07-16

**Authors:** Andrew J. Knighton, Ellen J. Bass, Elease J. McLaurin, Michele Anderson, Jennifer D. Baird, Sharon Cray, Lauren Destino, Alisa Khan, Isabella Liss, Peggy Markle, Jennifer K. O’Toole, Aarti Patel, Rajendu Srivastava, Christopher P. Landrigan, Nancy D. Spector, Shilpa J. Patel

**Affiliations:** 1 Healthcare Delivery Institute, Intermountain Healthcare, Murray, UT USA; 2 Department of Health Systems and Sciences Research, College of Nursing & Health Professions, Drexel University, Philadelphia, PA USA; 3 Department of Information Science, College of Computing and Informatics, Drexel University, Philadelphia, PA USA; 4 Health Systems & Sciences Research, College of Nursing & Health Professions, Drexel University, Philadelphia, PA USA; 5 Lucile Packard Children’s Hospital Stanford, Palo Alto, CA USA; 6 Institute for Nursing and Interprofessional Research, Children’s Hospital of Los Angeles, Los Angeles, CA USA; 7 St. Christopher’s Hospital for Children, Philadelphia, PA USA; 8 Stanford University, Pediatric Hospital Medicine, Palo Alto, CA USA; 9 Harvard Medical School, Boston Children’s Hospital, Boston, MB USA; 10 Walter Reed National Military Medical Center, Bethesda, MD USA; 11 Pediatrics and Internal Medicine, University of Cincinnati College of Medicine, Cincinnati Children’s Hospital Medical Center, Cincinnati, OH USA; 12 Rady Children’s Hospital San Diego, University of California, San Diego, CA USA; 13 Department of Pediatrics, University of Utah School of Medicine, Salt Lake City, UT USA; 14 Division of General Pediatrics, Boston Children’s Hospital, Boston, MB USA; 15 Sleep and Patient Safety Program, Brigham and Women’s Hospital, Boston, MB USA; 16 William Berenberg Professor of Pediatrics, Harvard Medical School, Boston, MB USA; 17 Boston Children’s Hospital, Boston, MB USA; 18 Executive Leadership in Academic Medicine (ELAM) Program, Drexel University College of Medicine, Philadelphia, PA USA; 19 Department of Pediatrics, Drexel University College of Medicine, Philadelphia, PA USA; 20 Kapiolani Medical Center for Women & Children, University of Hawaii John A. Burns School of Medicine, Honolulu, HI USA

**Keywords:** Patient and family-centered rounds, Multi-disciplinary teams, Structured communication, Implementation, Consolidated Framework for Implementation Research (CFIR), Barriers and facilitators, Mixed methods, I-PASS

## Abstract

**Background:**

Effective communication in transitions between healthcare team members is associated with improved patient safety and experience through a clinically meaningful reduction in serious safety events. Family-centered rounds (FCR) can serve a critical role in interprofessional and patient-family communication. Despite widespread support, FCRs are not utilized consistently in many institutions. Structured FCR approaches may prove beneficial in increasing FCR use but should address organizational challenges. The purpose of this study was to identify intervention, individual, and contextual determinants of high adherence to common elements of structured FCR in pediatric inpatient units during the implementation phase of a large multi-site study implementing a structured FCR approach.

**Methods:**

We performed an explanatory sequential mixed methods study from September 2019 to October 2020 to evaluate the variation in structured FCR adherence across 21 pediatric inpatient units. We analyzed 24 key informant interviews of supervising physician faculty, physician learners, nurses, site administrators, and project leaders at 3 sites using a qualitative content analysis paradigm to investigate site variation in FCR use. We classified implementation determinants based on the Consolidated Framework for Implementation Research.

**Results:**

Provisional measurements of adherence demonstrated considerable variation in structured FCR use across sites at a median time of 5 months into the implementation. Consistent findings across all three sites included generally positive clinician beliefs regarding the use of FCR and structured rounding approaches, benefits to learner self-efficacy, and potential efficiency gains derived through greater rounds standardization, as well as persistent challenges with nurse engagement and interaction on rounds and coordination and use of resources for families with limited English proficiency.

**Conclusions:**

Studies during implementation to identify determinants to high adherence can provide generalizable knowledge regarding implementation determinants that may be difficult to predict prior to implementation, guide adaptation during the implementation, and inform sustainment strategies.

**Supplementary Information:**

The online version contains supplementary material available at 10.1186/s43058-022-00322-1.

Contributions to the literature

- Identifies implementation determinants of high adherence to the use of an evidence based structured FCR in pediatric inpatient settings.
- Implementation determinants to high adherence, which include addressing those determinants that may be difficult to predict prior to implementation.
- These findings can help inform theory-driven strategies to implement structured FCR to achieve high adherence across diverse sites.

## Background

Miscommunications lead to medical errors and sentinel events (i.e., severe adverse events in hospitals) including patient injury or death [1, 2]. Effective communication in healthcare is associated with improved patient safety and experience [3–6]. Endorsed by the American Academy of Pediatrics (AAP) and the Institute for Patient and Family-Centered Care, family centered rounds (FCR)—multidisciplinary rounds conducted in patients’ rooms that integrate family members’ preferences in clinical decision making [7–9]—support inter-professional and patient/family communication [7]. The AAP policy provided limited information on how FCR should be specifically designed or implemented [7]. Despite stakeholder support and evidence of clinical and patient benefits [3, 10–12] adherence to FCR remains highly variable [9, 13].

Structured FCR approaches have been introduced to address barriers and facilitators (hereafter, determinants) to the use of FCR. Common structured elements of FCR established through a scoping review of relevant studies include the following: defined multi-disciplinary team roles on rounds, a defined location for rounds (usually at the bedside), an established rounding script or checklist to guide the care team presentation and interaction with the patient and family, and opportunity to incorporate and address nurse and family while developing a care plan [13]. Growing evidence suggests that more structured approaches to FCR are associated with increased clinician-family engagement in clinical decision making [14–16], more efficient rounds [17], improvements in overall nurse and patient satisfaction [18, 19], and an almost 40% reduction in harmful medical errors [3].

While demonstrating benefits, introducing more structured approaches such as FCR in complex adaptive system environments may introduce unique implementation challenges given the clinicians’ interactions with the intervention in the actual host context [20]. Understanding implementation determinants to high adherence, including those determinants that may be difficult to predict prior to implementation, is needed. The purpose of this study was to identify intervention, individual, and contextual determinants of high adherence to common elements of structured FCR in pediatric inpatient units during the implementation phase of a large multi-site study implementing a structured FCR approach.

## Methods

We performed an explanatory, sequential mixed methods study (quant -> QUAL) from September 2019 to October 2020: (1) to provisionally measure variation in adherence to common elements of a structured FCR approach across 21 pediatric inpatient sites covering all four regions of the USA (West, Midwest, South, and Northeast) during implementation of a national, multi-site project, and (2) to conduct qualitative interviews with clinicians at three sites to understand reasons for variation and identify organizational determinants to high adherence to structured FCR use [21, 22]. The research protocol was approved by Boston Children’s Hospital Institutional Review Board, the lead site for the larger dissemination and implementation study. We adhered to published best practices for reporting of mixed methods studies [22, 23] and qualitative research [24].

### Clinical intervention and implementation

Patient and Family Centered I-PASS Safer Communication on Rounds Everytime (I-PASS SCORE): Patients, Families, Nurses and Physicians Co-Producing Safer Care is a structured FCR approach with common elements to many structured rounding approaches. I-PASS SCORE elements include the following: (1) presence of a multi-disciplinary care team, (2) nurse sharing, (3) care team and family participation in overall and daily plans, (4) family and care team integration in the development of the daily care plan, (5) family engagement in specific actions of care, and (6) eliciting teach-back from the family to confirm understanding (see Table 1). Introduction of this structured approach in a prior 7-site study of pediatric patients led to a 38% reduction in reported serious harmful errors [3]. For the present study, the 21 pediatric sites had familiarity using FCR prior to the study though none had implemented a structured FCR approach like I-PASS SCORE. Settings within each site were selected where local site leaders determined that support and/or need for the intervention was high.

**Table 1:** Structured elements of the I-PASS SCORE family-centered rounds intervention [3]

Element	Element title	Element description
I	Introductions Illness severity - Better - Worse - Same	*• Multidisciplinary care team* presents at the bedside *•* Team members are introduced *•* Family asked if patient is better, worse, or the same as prior day. Parent asked what specific concerns or questions they would like addressed
P	Patient summary	*• Nurse invited to share* first on observations, questions, concerns, advocate for family *•* Physicians and other team members share observations and assessments using communication best practices *• Discussion occurs among team members* (including family) regarding clinical status, overall and daily treatment plans
A	Action list	*• Plans for the day* and are reviewed with *other team members* (e.g., clinical pharmacist, medical social worker), including family
S	Situation awareness/contingency plans	*•* If/then statements guide actions for potential future events (including major unexpected events) *• Family is engaged in specific actions of care and awareness* (e.g., “if there is a fever, we have to draw more blood cultures”)
S	Synthesis by receiver	*•* Physician elicits teach-back from family (e.g., “Would you mind sharing your understanding of the plans for the day?”, “When you speak with grandma later today, how will you describe what’s happening with José here at the hospital?”) *• Clarifications made as needed* to confirm shared mental model *•* Physician re-confirms plans with nurse

Italicized language highlights elements of I-PASS SCORE family-centered rounds (FCR) with common elements to many structured rounding approaches

The intervention was deployed across the 21 sites. At the program level, a national coordinating council led by the study principal investigator had primary oversight for intervention and measurement development, education and training material development, overall implementation and study execution, data collection and consolidation, and dissemination and reporting of study results. The national program was also responsible for the development and training of external physician, nurse, and family mentors that were assigned to a given site to provide mentored implementation support using a model adapted from the Society of Hospital Medicine Mentored Implementation approach [25]. At the site level, each site had a similar local leadership trio, including a physician, nurse, and family lead. The site leadership team was responsible for all aspects of local site implementation including local stakeholder engagement, deployment of education and training materials provided by the national-level program to front-line teams, local data collection, and efforts to identify and address local barriers to implementation (see Supplemental Table 1 and Supplemental Figure 1).

Each study site had a separate start date for adoption of the intervention following a baseline measurement period of at least 3 months and local education and training of affected teams using materials developed at the national program level. Following site adoption, each site measured performance for a 12-month intensive implementation period, while the national site mentor trio met routinely (at least monthly) with the local site leadership trio to review stakeholder engagement at the site, monitor performance, and work with local sites to identify and address ongoing barriers to adherence. Questions regarding site level intervention adaptation acceptability were brought to the national coordinating council for discussion with the intent to understand and support the local environment while retaining fidelity to the intervention structure. This 12-month implementation period was followed by a 6-month “graduated independence” period for each site. In graduate medical education, graduated independence describes the gradual lifting of supervision as the student or resident physician demonstrates competence in various clinical knowledge, skills, and attitudes in preparation for unsupervised clinical practice after graduation [26, 27]. In the context of this study, it reflects the shifting of responsibility of program-level tasks to the local site leaders with the goal of establishing site-level self-sufficiency and sustainability. During the graduated independence period, each site continued the intervention and set sustainment strategies upon study completion. Interviews were specifically timed to coincide with the intensive implementation period at each site.

### Provisional measures of site adherence for interview site selection

Variation in adherence to structured FCR was evaluated using two provisional measures as of July 22, 2020, at a median time of 5 months [range: 1–9 months] across sites following adoption of the structured FCR intervention at the site. As of July 22, 2020, all 21 sites had adopted the intervention and were working to identify existing and emerging determinants to promote increased uptake to and routine use of the structured FCR components. Two provisional measures of site level adherence to I-PASS SCORE based on direct rounds observations performed on a convenience sample of three patient rounding encounters per week were used to evaluate ongoing encounter-level uptake of the practice. Observations were conducted by a trained clinician using a direct observation tool with clearly described observable behaviors for the FCR components and assessment of level of participation (e.g., present but not paying attention; answers questions; asks questions; and speaks up to fill in missing details). The two site level measures were as follows: (1) estimated percentage of observed encounters where common structured FCR components were performed and (2) estimated percentage of observed encounters where the nurse was “almost always”/“always” included in the discussion by the presenter. The common structured FCR elements are listed in Table 1. We calculated a relative ranking by site for each provisional measure and generated an aggregate mean site ranking. Sites were divided into three terciles representing relatively high, moderate, and low-adhering sites. One site was selected from each tercile to participate in qualitative interviews.

### Tailored interview guide development

We developed an interview guide using a deductive, multi-method approach: a scoping review [13, 28–31] to examine general determinants to FCR use; a national technical expert panel of 11 participating healthcare institutions from the central program that included 18 physicians, 3 nurses, 3 patient/family representatives, 2 health services researchers, 1 human factors engineer, and 1 implementation scientist to identify existing or suspected barriers to implementation (simultaneous triangulation) [32]; and categorization of findings according to the Consolidated Framework for Implementation Research (CFIR) [33, 34] by an experienced implementation scientist (AJK). This approach is consistent with efforts to develop contextual implementation frameworks for complex system interventions [35, 36].

CFIR constructs identified as relevant for analyzing organization determinants through the scoping review and multidisciplinary expert panel formed the theoretical basis for the interview guide questions. Given our emphasis on understanding clinician interaction with the intervention in the host environment, the interview guide included the following in-scope CFIR domains: (1) individual (knowledge and beliefs, individual stage of change, other personal attributes); (2) intervention (relative advantage, complexity, evidence strength, adaptability); and (3) inner setting (relative priority, leadership engagement, compatibility, available resources, tension for change). The outer setting and implementation domains were excluded given the study scope. Validated interview questions were then drawn from the “Barriers to Physician Adherence to Practice Guidelines” model to guide assessment of knowledge, attitudes, and behaviors of sites and individual caregivers regarding more structured FCR approaches [37]. Validated questions were adapted from the Unified Theory of Acceptance and Use of Technology to explain user intentions for I-PASS SCORE and subsequent usage behavior [38]. Upon completion, the interview guide questions were reviewed for clarity and relevance by the study Scientific Oversight Committee including the study principal investigator and co-investigators, and members of the study Family Advisory Council, and then piloted with 2 clinicians. The piloted interviews were not included in the final analysis.

### Key informant semi-structured interviews

To understand site variation in adherence [22, 39], key informant semi-structured interviews were conducted with site clinicians. To ensure an information rich sample [40], the research team selected three sites using a stratified, purposive sampling approach [41] based upon site adherence relative ranking (high-medium-low). Other factors that were incorporated into the final site selection decision within each tercile included: pediatric hospital type (academic versus community), US geographic region (West, Midwest, South, Northeast), time from implementation go-live date to interviews, and local leadership support to participate in the study. Local leadership support was determined through conversations between the study team and local site leaders.

Two-person teams of trained, experienced qualitative researchers (AJK, EB, EM) conducted videoconference interviews with a purposive sample using a role-based criterion to ensure a representation of team roles until site thematic saturation was achieved (7–9 key informant interviews per site). Interview participant roles were outlined with a goal to achieve uniform participation at each site and included typical front-line rounding team members such as attending physicians, physician learners (resident physicians and medical students), nurses, site administrators familiar with the project, and project leaders. Local study site leads invited participants via email or direct conversation. They identified individuals within each role that varied in terms of years of experience and attitudes and beliefs regarding FCR. The study team conducted assessment of thematic saturation using field notes following site completion of interviews. All interviews were recorded, transcribed, and de-identified for subsequent coding and analysis.

### Qualitative data analysis

A hybrid qualitative content analysis paradigm was applied to interview data, incorporating open-iterative and directed approaches to identify determinants to the implementation of structured FCR [42]. The codebook was organized by interview guide question as the unit of analysis and incorporated codes for each interview guide question were derived from a sample of the text data by question and from pre-determined response codes. Two investigators reviewed the preliminary codebook to ensure face validity and completeness prior to coding.

The coding was performed by four investigators (AJK, EB, EM, SP) organized into two dyads. The coding was done in two phases. In the first phase, each dyad coded half the transcribed interviews. Within each dyad, each reviewer independently read and coded each transcript using the codebook. New codes were added based on consensus from both reviewers in a dyad. Within each dyad, discrepancies were discussed until consensus was reached. During the second phase, the two dyads conducted sub-theme analysis on half of the individual codes. A final assessment of thematic saturation by site was conducted by the four investigators. Reported implementation barriers and facilitators were summarized according to the CFIR framework [33]. Comments regarding the implementation and outer setting constructs were excluded from the final presentation of results at this stage. Verbatim transcript quotations were adapted into readable communication, as needed, for manuscript presentation [43, 44]. Coding was done in Microsoft Excel for Office 365 (Microsoft Corporation, Seattle, WA, USA).

## Results

Provisional measurement of the site percentage median with all six common structured FCR elements observed was estimated at 40% [range: 20–60%] based upon more than 2500 specific rounding encounter observations performed across all sites as of the measurement date. The site percentage median with nurse inclusion by the presenter in the rounding discussion was estimated at 60% of the time observations were conducted [range: 30–100%]. Site relative ranking, along with individual site percentage above/below the site median for each provisional measure, is included in Table 2.

**Table 2:** Provisional measurement results for site selection

		Percentage over (+)/under (−) site median	
Site rank	Time since adoption of structured FCR Intervention (in months)	Structured family-centered rounds components observed	Nurse inclusion by presenter on rounds observed
1	2	+ 50%	+ 67%
2*	2	+ 50%	+ 17%
3	7	0%	+ 25%
4	8	0%	+ 25%
5	1	0%	+ 25%
6	7	− 13%	+ 25%
7	5	0%	+ 17%
8	7	0%	+ 17%
9	7	0%	0%
10	7	− 25%	17%
11	1	0%	− 8%
12	1	0%	− 8%
13	1	13%	− 17%
14*	9	− 13%	− 8%
15	5	− 38%	0%
16	7	− 38%	− 25%
17	6	− 25%	− 33%
18	3	− 13%	− 50%
19*	7	− 38%	− 33%
20	3	− 50%	− 50%
21	1	− 50%	− 50%

The (*) denotes sites selected for field interviews. Overall median time since adoption was 5 months. Median site adherence to structured family-centered rounds (FCR) components and nurse participation observed was 40% and 60%, respectively. Common structured FCR components observed are listed in Table 1

### Qualitative findings on FCR and I-PASS SCORE practices

Twenty-four key informant interviews were conducted at three sites. The majority of interviews were 30 min (range: 15–40 min). Participant roles by site are shown in Table 3. Saturation was reached at each site. No site utilized a scheduling coordinator for rounds or established a fixed time schedule for each patient visit. The timing and sequencing of rounds was driven primarily by the attending physician. Organizational determinants follow, organized by CFIR domain with complete results listed in each table: individual, intervention, and inner setting.

**Table 3:** Key informant interview participant roles by site

Role	High-adhering site	%	Moderate-adhering site	%	Low-adhering site	%	Total	%
Attending physicians	3	33	3	37	1	14	7	29
Learners (interns, residents, students)	4	45	1	13	1	14	6	25
Nurses	1	11	2	25	2	29	5	21
Site administrators/project leaders	1	11	2	25	3	43	6	25
Total	9	100	8	100	7	100	24	100

### Implementation determinants—individual CFIR domain (Table 4)

Clinicians at all three sites expressed strong beliefs that structured FCR approaches like I-PASS SCORE can provide observable benefits that increase family satisfaction through empowerment and engagement in their child’s care.

I think [I-PASS SCORE] improves the patient experience, period…if they realize that we’re all on the same page here and we have the best interests of their child in mind, I think that improves their satisfaction with the care. -Attending, high-adhering site

We’re held more accountable…by making sure that the family understands…why they’re there, what we’re doing, and what has to be done before they leave. Those are things, I think, that have gotten blurred in the past…It just seems more complete. – Nurse, low-adhering site

So many [patients and families]…have appreciated that people…talk to them about their child and ask for their input, and to consider them a partner in their child’s care. I do really think families appreciate it. -Attending, moderate-adhering site

**Table 4:** Qualitative content analysis of key informant interviews—individual domain of the Consolidated Framework for Implementation Research (CFIR)

***Individual domain*** qualitative content analysis themes	
***Facilitators***	***Construct***
Relatively consistent beliefs regarding which components of family-centered rounds (FCR) are most important	Knowledge and beliefs
Belief that structured FCR approaches like I-PASS SCORE increase care team and family understanding of the care plan	Knowledge and beliefs
Belief that structured FCR approaches like I-PASS SCORE provides a forum for families to express concerns and to feel engaged and empowered to participate in their child’s care	Knowledge and beliefs
Belief that structured FCR approaches like I-PASS SCORE demonstrate respect for the family’s role and increases family awareness of the healthcare team and their connection to the team	Knowledge and beliefs
Belief that structured FCR approaches like I-PASS SCORE provide a structure for rounds that benefits the care team, including resident learners	Knowledge and beliefs
Belief that structured FCR approaches like I-PASS SCORE take longer but efficiencies are gained throughout the day with fewer follow up questions regarding the plan	Knowledge and beliefs
Belief that structured FCR approaches like I-PASS SCORE increase patient and family overall satisfaction with care	Knowledge and beliefs
Belief that structured FCR approaches like I-PASS SCORE improve care outcomes	Knowledge and beliefs
Participants express a high level of confidence in their ability to follow structured FCR approaches like I-PASS SCORE	Self-efficacy
Some individuals intend to use structured FCR approaches like I-PASS SCORE going forward in their practice	Stage of change
Participants have high confidence that the organization will continue to use structured FCR approaches like I-PASS SCORE when the project is complete	Stage of change
***Barriers***	
Variation in beliefs about the purpose of rounds across stakeholders	Knowledge and beliefs
Physician with more experience may be less willing to adhere to more structured approaches.	Personal attributes
Nurses may not believe their attendance on rounds is a priority for the team (e.g., they are not actively included in rounds) and not a good use of their time.	Knowledge and beliefs
Nurses believe they can address questions regarding the care plan with physicians more efficiently at other times	Knowledge and beliefs
Family cultural norms (role of authority figures, et al.) effect their level of participation during rounds	Personal attributes
Presenters/learners do not want to appear “wrong” in front of peers and families	Self-efficacy
Belief by some participants that family presence and emphasis on use of plain language can limit content discussed during rounds.	Knowledge and beliefs

List of frequent or compelling individual determinants identified through field interview to organization implementation of a structured FCR approach

Participants from the high-adhering site noted that more structured approaches to FCR can align closely with a healthcare organization’s broader goals for patient care.

We have a huge responsibility because we care for people here regardless of their ability to pay … the I-PASS [SCORE]…falls into that mission. It’s taking…the information and…honing it and carving…in a way that they’re including the family and their challenges. I think they certainly can and will [continue to use I-PASS SCORE] because I think that it has built a structure into the rounds that…has added to the whole conversation about the patient... -Nurse Administrator, high-adhering site

Moderate and low-adhering sites noted variation in perceptions regarding the purposes of rounds persisted across stakeholders despite implementation of the intervention.

Another challenge … is just that we’re not all on the same page about the purpose of rounds, especially when it comes to teaching. … We may talk about some details with the team or just with the senior outside of the room and do…what’s appropriate in the room…but some of us want to hear about every single lab value in the room and have the team go over it, so we have a bunch of work to do there. -Attending, moderate-adhering site

Participants at moderate and high-adhering sites expressed that a structured FCR approach like I-PASS SCORE empowers resident and student learners to engage in rounds with greater self-efficacy by providing a clear format for rounds.

A template like I-PASS…helps you remember to stay on top of everything that you need to share with a family, making sure that the family is attended to properly. … It is especially helpful for the medical students who…don’t really know how to have these kinds of conversations… -Intern, high-adhering site

However, this did not fully address persistent concerns across all sites regarding learner self-efficacy to present in front of families, and the belief that family presence and plain language can restrict the content discussed during rounds.

*…*we don’t talk as in-depth as we normally [would]…because we don’t want to confuse the families and … say things that we don’t actually think the diagnosis is…we don’t have as much discussion expanding our…differential…we don’t talk about…scary contingencies…because we are trying to protect…the families and patients from hearing… those things. -Nurse, low-adhering site

What [students] don’t like is that they have to modify their presentation about the patient to fit the living-room language…to fit having the family there. They don’t like the feeling of possibly being what they consider to be “corrected”…because they have a sense that they lose credibility with the family. -Attending, moderate-adhering site

Participants from higher adhering sites also noted that a structured FCR approach like I-PASS SCORE may take longer to perform than historic FCR methods used, but they believe that efficiencies are gained with fewer follow up questions throughout the day.

I mean if you could get everyone at the same time in the same place once, that certainly improves your day from a clinical standpoint, opens up time for other activities, whether it’s teaching, whether it’s more time at the bedside…-Attending, high-adhering site

If the rounds are good, I wouldn’t have to call the doctors about very much, which is way easier on my job than if you don’t have good rounds and I have to call about everything. -Nurse, moderate-adhering site

Nurse attendance and engagement in rounds remain a persistent challenge. Nurse engagement was impacted by beliefs by some that participation in rounds was not a good use of nursing time and that when nurses do attend, they may not be actively invited to participate. Alternatively, nurses may prefer to ask questions regarding the care plan with physicians at other times (e.g., catching them in hallway) rather than having to attend the full rounding encounter.

…they were getting the nurse there…and then no one was talking to the nurse until the end, sort of in passing after the plan has already been made. And one of the nurses told me “Oh, don’t worry, we would chime in if there was an issue.” I think that’s true of some of the nurses, but some of our newer nurses are more reluctant to speak out especially in front of a large team…but will talk to the team afterwards. That sort of defeats the purpose of constructing that whole plan that then is potentially going to get altered with some additional information. -Attending, moderate-adhering site

### Implementation determinants—intervention CFIR domain (Table 5)

When asked to recall I-PASS SCORE elements to assess the participant familiarity with and underlying complexity of the structured FCR intervention, clinician interview participants across all three sites could frequently recall common intervention elements including presence of a multidisciplinary team and introductions, having the family speak, nurse participation, shared care plan development, capturing family feedback, and understanding of the care plan and the use of plain language. While considered important as a communication method by many, assessing family understanding of the care plan was not consistently performed by clinicians as many family members were often intimidated when asked to teach back the plan for the day. This was particularly challenging when language barriers to communication were present.

…sometimes I still feel like family members are a little bit…startled when we ask them to do the teach-back…I noticed it’s the one part that…everybody pretty much across the board in our program doesn’t do as much. -Resident, low-adhering site

**Table 5:** Qualitative content analysis of key informant interviews—intervention domain of the consolidated framework for implementation research

***Intervention domain*** qualitative content analysis themes	
***Facilitators***	***Construct***
Sites continued to utilize I-PASS SCORE during the COVID-19 pandemic with modest adaptations or adjustments	Adaptability
Learners find participating in structured FCR approaches like I-PASS SCORE valuable for professional development	Relative advantage
***Barriers***	
Adhering to the order of presentation on structured FCR like I-PASS SCORE is not always ideal for answering a family’s questions	Adaptability
Learners have difficulty using plain language on rounds	Complexity
Participants have difficulty articulating a connection between structured FCR approaches like I-PASS SCORE and patient health outcomes	Relative advantage
Learners have a difficult time diplomatically soliciting teach back or collecting feedback	Complexity
Lack of clear parameters for defining acceptable, evidence-based variation using structured approaches like I-PASS SCORE make adaptation difficult	Adaptability
The existing structured FCR intervention design requires a new paradigm for teaching and learning at the bedside.	Complexity
Sites had difficulty operationalizing methods for adhering to the structured FCR approach with a limited English proficient family requiring interpreter services	Complexity

List of frequent or compelling intervention determinants identified through field interview to organization implementation of a structured FCR approach

Using plain language when communicating on rounds remains difficult for some learners accustomed to using clinical language on other rotations.

I feel like it’s a culture that some medical students feel like they need to use fancy words …and while I do think that there’s some role for that like when you’re talking doctor to doctor, I think that there needs to be a little bit of a culture shift…If your goal is for the family to understand what you’re saying, then using [fancy] words makes no sense whatsoever. -Attending, high adhering site

Consistent with many structured interventions like I-PASS SCORE, site-level adaptability was a common theme across all sites. High- and moderate-performing sites maintained adherence to this structured FCR approach during the COVID-19 pandemic with modest adaptations, most notably with the use of telehealth solutions to enable remote rounds participation.

I’ll call it…social-distance rounding…we’ve been just having either the attending by themselves or usually it’s the attending and the senior-level resident go around with an iPad, and then the rest of the medical team is in one of the conference rooms [with another iPad/on the computer]…-Resident, moderate-adhering site

A common struggle across sites was adapting the structured FCR approach effectively for families with limited English proficiency (LEP) (e.g., when interpreter services were required).

We never really addressed the LEP [Limited-English Proficient] families and what kind of workflow we would standardize or anything like that...That is something that’s not really been studied so far, and so I think looking at how we interact with our LEP families here and trying to figure out what are we finding from the results. -Project Leader, high-adhering site

### Implementation determinants—inner setting CFIR domain (Table 6)

Leadership support was described as a facilitator at all three sites to the use of structured FCR approaches. At the high-adhering site, leadership support came in a well-articulated linkage that emerged between intervention use and the mission of the organization. Team members articulated a clear and consistent value statement when using a more structured FCR approach like I-PASS SCORE.

They…have asked us to come once or twice to present to the CEO [chief executive officer], the CMO [chief medical officer], the CNO [chief nursing officer]….Things that have actually been in place that have helped us…get things done is that we have a department of research across both our hospital and our sister institution… in pediatrics [they] meet pretty frequently and support [research] projects. -Attending, high-adhering site

**Table 6:** Qualitative content analysis of key informant interviews—inner setting domain of the consolidated framework for implementation research

***Inner setting domain*** qualitative content analysis themes	
***Facilitators***	***Construct***
Structured FCR approaches like I-PASS SCORE align with the values and culture of the organization	Culture
Leadership support for structured FCR is strong with physician, nurse and patient experience leaders	Leadership support
***Barriers***	
Sites stopped doing certain structured FCR components in response to the COVID-19 pandemic	Implementation climate
Lack of standardization in approach despite the presence of a structured FCR approach like I-PASS SCORE across attendings	Compatibility
Subspecialists often make care plans without including the rest of the patient care team	Networks and communications
Nurse participation on rounds is often subject to availability and competing patient priorities during rounding times	Compatibility
Structured FCR approaches like I-PASS SCORE disrupt existing clinical workflows requiring redesign, particularly for nurses	Compatibility
Nurse staff not properly resourced to support structured FCR approaches like I-PASS SCORE	Available resources
Nurses utilize workaround methods to accomplish the goals of structured FCR while limiting their time participating	Compatibility
Ongoing schedule changes lead to regular adjustments in team composition that impact rounding team development and performance	Compatibility
Rounding schedules may not allow families to attend rounds	Compatibility
Lack of resources as well as cultural barriers limit participation of limited English proficient families during rounds	Available resources

List of frequent or compelling inner setting determinants identified through field interview to organization implementation of a structured FCR approach

The moderate and low-adhering sites described a more bottom-up approach to practice dissemination of structured FCR methods originating largely in the efforts of a locally organized project team led by a few key individuals engaging leadership for support and executing most project requirements.

I think one thing we forget to do when we agree to do these large projects is to get that buy-in, and I would say that at [this site], you know, the opportunity came up, and the project lead decided, you know, “Yes, I want to do this,” you know, but I don’t think [they] ever brought it to a division meeting…I think we could’ve done more of that, like let’s be sure that we really are ready to do this, that we really want to do this. Attending, moderate-adhering site

Nurse attendance and nurse participation on rounds remained a structural barrier to full adherence to structured FCR at all three sites. Workflow disruption, including the need to adjust the timing and sequencing of activities, including patient priorities, was a primary barrier to nurse attendance and participation.

So far it’s…rearranging the times of the rounds so that it’s more in line with how the nurses are able to be present for the rounds, and…being respectful of their time and…being on time. – Nurse, high-adhering site

I think the nurse inclusion part is where we fail the most, and not that it’s the doctor’s fault. They all round approximately at the same time. You have three teams here rounding, and I’ve been there, and we try really hard, and the nurses really do want to be involved in the rounds, but, you know, it’s always in the morning where it’s difficult for us. -Nurse, low-adhering site

Often our teams aren’t always good about giving [nurses] advance notice…it is often only a few minutes [before rounds begin] and if you’re gowned and in a room taking care of a patient, that’s really hard to drop everything that you’re doing to get to this other room for rounds…that’s our biggest barrier. -Attending, moderate-adhering site

… they had the nurse in there, they did introduce the nurse. I don’t think that they ever discussed anything with the nurse. The nurse left about three-quarters of the way through, and I didn’t blame the nurse. They weren’t including her. She’s got things to do… -Nurse, low-adhering site

Further, the fact that many nurses were not attending rounds consistently in the past added incremental time commitments to their day that were not resourced prior to implementation.

[The nurses] want to get going, and, you know, rounds…can take up to 10 minutes, and I’ve been here and had three patients or four patients…[physicians are] all rounding, boom, you know, one after another (if you’re lucky one after another), sometime all at the same time because they’re different teams. It’s a big chunk of your time, and now all of a sudden, you’re behind. -Nurse, low-adhering site

Many nurses resort to workarounds to balance competing priorities.

I think it’s hard for [nurses] because they are trying to round when sometimes it’s right when meds are due, and I know they’re trying – I think some of them think if they’re in the room, they can go ahead and check things off their computer. They’re not always listening or participating. – Attending, low-adhering site

We talk to our nurses pretty consistently about what’s going on in rounds and how is it going because we see our nurses pop in, pop out. They’re [never] in the room beside the bed where they should be because they’re always like, “I already told the doctors what my problems were before they went into the room, and I really just want to be there for the plan of care...” I said, “But do you think it’s good for the family to hear, the parents to hear, what you’re telling the doctors about what you feel is important for their care?” and they’re like, “Well, yeah, but I’ve usually already talked to the parents and said, ‘I brought up to the doctors this, this, this, and this,’” so it’s not necessarily part of rounds. It’s the pre-rounding part. -Nurse leader, moderate-adhering site

## Discussion

Studies conducted during implementation to identify determinants to high adherence can provide generalizable knowledge regarding determinants that may be difficult to anticipate prior to implementation, guide adaptation during the implementation, and inform sustainment strategies. While structured approaches to FCR rounds may prove useful in addressing certain limitations in implementing FCR, certain barriers persist. Consistent findings across three sites with varying adherence levels include generally positive clinician beliefs regarding the use of FCR and structured rounding approaches, benefits to learner self-efficacy through structured approaches, potential efficiency gains derived through greater rounds standardization, as well as persistent challenges with nurse presence and engagement on rounds, and coordination and use of resources for LEP families.

The presence of widespread, positive clinician beliefs regarding the benefits of structured FCR upon implementation are consistent with other studies noting positive clinician attitudes and beliefs regarding the goals of FCR more generally, including nurse and family engagement in rounds [9, 45–52]. The high adhering site was effective at harnessing these beliefs to establish and situate an emerging positive narrative for using structured FCR within the dynamic properties of their context as a safety net hospital in an urban center [53, 54]. An administrator at the high-performing site noted enabling communication via structured FCR also facilitated engagement with families on other social needs that many healthcare organizations seek to address such as identifying housing or food insecurity concerns that may impact care plans [53–56].

Structured FCR approaches like I-PASS SCORE did not fully negate student and resident self-efficacy concerns when presenting in front of families. These concerns are consistent with earlier studies that physician learners feel that FCR can impede learning, increase learner fears about losing patient respect if they do not demonstrate having total knowledge, resulting in loss of patient/family trust [45, 50, 57, 58]. However, participants in our study noted a more formal, structured rounds approach that defined clear roles and set standard operating procedures for rounds was useful to learners when interacting with the rounding team. Given that rounds practices were usually driven by the attending physician and approaches and styles differed meaningfully, learners expressed that introduction of a more standard approach applied consistently by attending physicians within a unit helped them understand what to expect during rounds and defined more clearly their role on rounds.

Introduction of a structured FCR approach has the potential for efficiency gains. FCR studies show an increase in rounding length per patient per day by an average of 1.0–1.5 min [59, 60], though study results vary [15, 61]. The prior seven-center effectiveness study implementing structured FCR using I-PASS did not show a significant increase in rounding time [3]. Consistent with Kipps, et al. [60], we found that participants at the high and moderate adhering sites felt that any increase in structured rounds length early in the day had positive effects later in the day, reducing the overall average time spent per patient throughout the day and thus increasing overall efficiency. Further research should measure changes in the daily time spent on related communication for each patient.

Incorporating structure into FCR does not necessarily facilitate nurse attendance and participation in rounds. Workflow disruption, including the need to adjust the timing and sequencing of activities, including patient priorities, remains a determinant to high nurse attendance and participation consistent with other studies [9, 17, 46, 62–65]. Organizations should acknowledge that the implementation of structured FCR requiring nurse attendance as an organization standard can introduce additional nurse work requirements and may require changes in resource staffing during resource-strained periods [61, 63]. When making these adjustments, organizations should work to ensure that when nurses attend they are enabled to interact with the care team, contribute meaningfully to development of the daily care plan, advocate for patient/family needs and concerns, and are listened to.

Coordination and use of resources for families with LEP also remain a persistent challenge. Like other FCR studies [66, 67], a meaningful percentage of sites in the study had a significant number of patients with LEP and, upon implementation, struggled with the interaction between the clinical care team, interpreter, and family. Workflow evaluation with interpreters should establish an optimal approach to clinician-interpreter-patient interaction during structured FCR [68, 69]. Telehealth usage, made more routine through the COVID-19 pandemic response, shows promise for improved nurse and family participation and interpreter use [70].

Analysis of implementation determinants, often done before implementation, can be useful in informing initial implementation strategies a priori [36]. However, in complex system environments such as inpatient settings, the interaction of the clinical team and the intervention within a particular, often dynamic, host context, may limit the use of a priori assumptions to achieve high practice adherence [71–74]. We found that formally studying determinants a posteriori or during the implementation phase may be particularly useful for understanding high adherence determinants—including social interaction effects that are difficult to predict prior to implementation. For example, it can be difficult during initial implementation planning to understand how people will interact with an innovation in a complex environment with formal and informal workflows and information exchange that can impact adherence. The potential presence of these interaction effects may begin to explain why some evidence-based clinical practice implementations require multiple iterations to achieve high adherence [75]. Examples of interactions we identified a posteriori included the effects of nurse, interpreter, and learner interaction with the care team during rounds; nurse-physician interaction outside of rounds; and care team response to COVID-19 policies.

The results of this study are actionable and can inform follow-up implementation studies that measure the effects of specific strategies on adherence to structured FCR. Specific implementation strategies should (1) determine optimal physician and nurse workflows and staffing needs prior to implementation that accommodate role availability and participation; (2) establish and implement an optimal workflow for physician-interpreter-patient interaction; and (3) consider using enabling telehealth technologies to facilitate virtual coordination among the care team and family. These studies should also examine implementation workflow outcomes associated with intervention deployment, including efficiency and timeliness [76]. Study findings also highlight the potential benefits of conducting qualitative field work, once the implementation is underway, to reveal emerging determinants often difficult to anticipate, identify, and address in initial implementation planning. Future qualitative studies should seek to confirm the presence of emerging determinants across other evidence-based interventions in other settings.

This study has limitations. The study methods do not establish a clear causal relationship between specific implementation determinants and high adherence to structured FCR approaches such as I-PASS SCORE. Given the emphasis on individual, intervention, and contextual determinants and the availability of other sources on patient/family attitudes regarding FCR, we did not interview patients for this study. Patients are a vital component to successful introduction of structured FCR and more study is needed to overcome barriers to family participation. Some participant comments regarding determinants of structured FCR, including I-PASS SCORE, may have been referencing FCR more generally. We included two trained interviewers in each interview to better elicit understanding and to better clarify participant comments. Some variation in site level adherence may be explained by interrater observation differences at sites, though we trained reviewers using simulations and a standardized observation tool. Observation assessments were conducted weekly at each site beginning in the pre-implementation phase with more than 2500 observations completed as of the provisional measurement date across all sites. Some variation in provisional adherence measures may be explained by differences in implementation duration at the time of measurement as well as pre-implementation familiarity with FCR. To mitigate this, sites were selected where support and/or need for the intervention was high as determined by study and local leaders. All sites had adopted the intervention and site training was completed prior to the provisional measurement date and subsequent field work. Finally, we relied upon local site leadership to identify interviewees and limited the number at each site due to time and resource constraints. However, instructions were provided to local site leaders to identify individuals that varied in terms of years of experience and attitudes and beliefs regarding structured FCR. Evidence of saturation at each site also mitigated the risk that important perspectives were not captured. Further, the field interview team reviewed the site interview list during the study period to determine if additional individuals should be added.

This study had several strengths including the geographical scope of the study given unwarranted regional variations in pediatric care that suggest potential differences in implementation contexts that could vary the types of determinants identified [77, 78]. Other strengths include the methodological rigor of the study, including development of a theory-informed interview guide; the use of provisional performance data for site selection based upon a large observation sample; a sampling approach that ensured diverse viewpoints were represented across diverse settings; and interview timing during the intensive intervention period to understand emerging determinants during implementation to inform ongoing implementation and practice sustainment. These results can be used to develop a future hybrid implementation-effectiveness trial or other experimental or observational study to measure the effects of tailored implementation strategies that address identified determinants to structured FCR on adherence and related patient safety outcomes.

## Conclusions

Studies conducted during implementation to identify determinants to high adherence can provide generalizable knowledge regarding implementation determinants that may be difficult to predict prior to implementation, guide adaptation during the implementation, and inform sustainment strategies. Consistent findings across three sites of varying adherence to a structured FCR process include generally positive clinician beliefs regarding the use of FCR and structured rounding approaches, benefits to learner self-efficacy through structured approaches, potential efficiency gains derived through greater rounds standardization, as well as persistent challenges with nurse engagement on rounds, and coordination and use of resources for families with LEP.

## Supplementary Information

**Additional file 1:.** Supplementary Figure 1. Program-Site Coordination model for implementation of Patient and Family Centered I-PASS Safer Communication on Rounds Everytime: Patients, Families, Nurses and Physicians Co-Producing Safer Care (I-PASS SCORE). Each site had a local site leader trio including a family, nurse and physician lead responsible for site level implementation. Each site leadership trio was mentored by an assigned national mentor trio composed of a family, nurse and physician mentor with experience and training in the deployment of I-PASS SCORE.

**Additional file 2:.** Supplemental Table 1. Patient and Family-Centered I-PASS SCORE: Patient and Family Centered I-PASS Safer Communication on Rounds Everytime: Patients, Families, Nurses and Physicians Co-Producing Safer Care (I-PASS SCORE) program and site level implementation responsibilities.

## Data Availability

All provisional quantitative data generated or analyzed during this study is included as aggregated in the published article in Table 2. The majority of qualitative data for this study is made available in the publication tables. However, the detailed interview dataset generated and analyzed during the current study is not publicly available due to individual privacy concerns.

## Abbreviations

AE: Adverse event
CFIR: Consolidated Framework for Implementation Research
EHR: Electronic health record
FCR: Family-centered rounding or rounds
I-PASS: Illness Severity, Patient Summary, Action List, Situation Awareness and Synthesis mnemonic
I-PASS SCORE: Patient and Family-Centered I-PASS Safer Communication on Rounds Everytime: Patients, Families, Nurses, and Physicians Co-Producing Safer Care
LEP: Limited-English proficient or proficiency
HIT: Health information technology
QI: Quality improvement
