# Supplementary figures and images for: Intervention, individual, and contextual determinants to high adherence to structured family-centered rounds: a national multi-site mixed methods study
Source: Implement Sci Commun. 2022 Jul 16;3:74. doi: 10.1186/s43058-022-00322-1 (PMC9287702; doi:10.1186/s43058-022-00322-1)

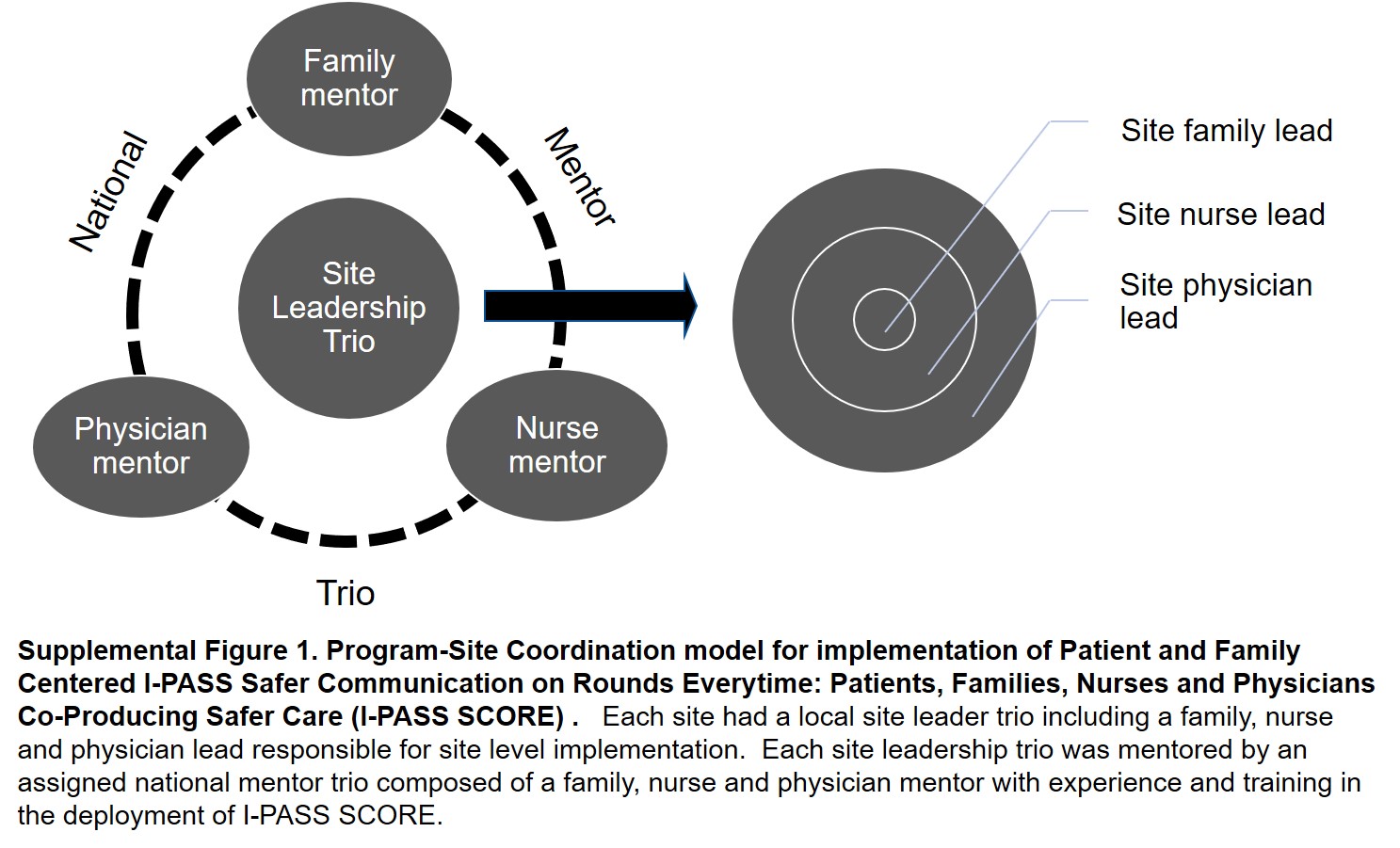

Supplement: Supplementary file 1 — Additional file 1:. Supplementary Figure 1. Program-Site Coordination model for implementation of Patient and Family Centered I-PASS Safer Communication on Rounds Everytime: Patients, Families, Nurses and Physicians Co-Producing Safer Care (I-PASS SCORE). Each site had a local site leader trio including a family, nurse and physician lead responsible for site level implementation. Each site leadership trio was mentored by an assigned national mentor trio composed of a family, nurse and physician mentor with experience and training in the deployment of I-PASS SCORE. [file 43058_2022_322_MOESM1_ESM.jpg]
