# Supplementary material for: Intervention, individual, and contextual determinants to high adherence to structured family-centered rounds: a national multi-site mixed methods study
Source: Implement Sci Commun. 2022 Jul 16;3:74. doi: 10.1186/s43058-022-00322-1 (PMC9287702; doi:10.1186/s43058-022-00322-1)
Supplement: Supplementary file 2 — Additional file 2:. Supplemental Table 1. Patient and Family-Centered I-PASS SCORE: Patient and Family Centered I-PASS Safer Communication on Rounds Everytime: Patients, Families, Nurses and Physicians Co-Producing Safer Care (I-PASS SCORE) program and site level implementation responsibilities. [file 43058_2022_322_MOESM2_ESM.docx]

**Supplemental Table 1. Patient and Family-Centered I-PASS SCORE: Patient and Family Centered I-PASS Safer Communication on Rounds Everytime: Patients, Families, Nurses and Physicians Co-Producing Safer Care (I-PASS SCORE) program and site level implementation responsibilities**

| **PROGRAM OVERSIGHT** | **MEMBERS^1^** | **RESPONSIBLE FOR…** |
| --- | --- | --- |
| **NATIONAL PROGRAM GOVERNANCE** | | |
| National Coordinating Council | 13 members | Primary oversight of the national implementation and study execution, dissemination and reporting, supported by national committees |
| Education and Training Committee | 14 members | Development of education and training materials |
| Mentorship Committee | 3 members | Development and delivery of national mentor support |
| Implementation Committee | 17 members | Design, development, and execution of implementation strategies and materials, to provide training and mentoring materials pertinent to implementation for national committees, national site mentors/trios, and local site leaders |
| Scientific Oversight Committee | 14 members | Primary oversight for the design and development of measurement tools and strategies, tools (surveys, questionnaires, etc.). Oversight of data, data governance, and dissemination to national and site level leaders |
| Family Advisory Council | 11 members drawn from previous Patient and Family Centered I-PASS Study Group and newly recruited members | Primary oversight for materials for patients and families (e.g., orientation brochure for family centered rounds). |
| Nursing Advisory Council | 4 members drawn from previous Patient and Family Centered I-PASS Study Group and newly recruited members | Primary oversight of materials pertinent to nursing orientation and education to the intervention. |
| National Mentor Trio(s)  See Supplemental Figure 1 for relationship between National Mentor Trio and Site Leadership Trio | National Family, Physician and Nurse Mentor with experience and training in deployment of Patient and Family Centered I-PASS SCORE | Guiding the local site leadership trio through the implementation of the intervention. This was accomplished through a regular cadence of meetings to:   - Review stakeholder engagement at the site - Review adherence via monthly run charts - Identify and address local barriers to implementation |
| **LOCAL SITE GOVERNANCE (Each of 21 sites)** | | |
| Site Leadership Trio  See Supplemental Figure 1 for relationship between Site Leadership Trio and National Mentor Trio | Site Family, Physician and Nurse Lead/Representative at each site | Site-level implementation of the intervention, working respectively with patients/families and front-line physicians and nurses, including:   - Local stakeholder engagement - Dissemination of education and training materials - Rounds observations (performed weekly) - Collection of surveys from families, other stakeholders (e.g., learners, nurses, attending physicians) - Identification and resolution of local barriers to use |

^1^All national committees had family, nurse and physician representatives.
